# Choroidal alterations of Sturge-Weber syndrome secondary glaucoma and non-glaucoma port-wine stain patients distinguished by enhanced depth imaging optical coherence tomography

**DOI:** 10.1186/s12886-020-01744-y

**Published:** 2020-12-07

**Authors:** Yue Wu, Lulu Huang, Yixin Liu, Li Xu, Wenyi Guo

**Affiliations:** 1 Department of Ophthalmology, Ninth People’s Hospital Affiliated to Shanghai Jiao Tong University School of Medicine, No. 639 Zhizaoju Road, Huangpu District, Shanghai, 200011 China; 2 Shanghai Key Laboratory of Orbital Diseases and Ocular Oncology, Shanghai, 200011 China

**Keywords:** Sturge-weber syndrome, Port-wine stain, Glaucoma, Choroidal Hemangiomas, Optical coherence tomography, Complications

## Abstract

**Background:**

To evaluate the choroidal changes in Sturge-Weber syndrome (SWS) secondary glaucoma and non-glaucoma port-wine stain (PWS) patients by enhanced depth imaging optical coherence tomography (EDI-OCT).

**Methods:**

SWS and PWS patients who were over 3 years old and treated or screened at our ophthalmology department were included in the study. Baseline demographics, EDI-OCT and fundus photography data were collected from all patients.

**Results:**

Overall, 46 non-glaucoma PWS (NGPWS) patients and 35 SWS secondary glaucoma (SG) patients were included, with mean ages of 16.52 ± 13.63 and 13.94 ± 8.27 years, respectively (*p* > 0.05). Among these patients 2 exhibited bilateral PWS and unilateral glaucoma. Thus, the two eyes of each patient were divided into NGPWS and SG group, respectively. Twenty-one eyes had choroidal hemangiomas and 7 eyes had excessive thickening of the choroid without choroidal hemangiomas. Choroidal hemangiomas were only observed in ipsilateral eyes of SG patients. The choroidal thicknesses of the ipsilateral and fellow eyes of NGPWS patients were 358.10 ± 117.40 μm (45 eyes) and 288.20 ± 79.04 μm (41 eyes), respectively (*p* < 0.05). The choroidal thicknesses of the ipsilateral and fellow eyes of SG patients were 511.40 ± 242.10 μm (15 eyes) and 283.90 ± 92.27 μm (29 eyes), respectively (*p* < 0.05). Significant differences were found between the ipsilateral eyes of SWS and PWS patients (*p* < 0.05). Six of 13 eyes (46%) with choroidal hemangiomas exhibited post-operative posterior segment complications.

**Conclusions:**

NGPWS and SG patients had a thicker choroid in the ipsilateral eye. The trend was even more pronounced in SG patients. Choroidal hemangiomas were only found in the ipsilateral eyes of SG. In addition, choroidal hemangioma was a risk factor for post-operative posterior segment complications in SWS patients.

## Background

Sturge-Weber Syndrome (SWS) is a rare, sporadic, congenital, neurocutaneous disorder with angiomas characterized by cutaneous capillary malformations (port-wine stain [PWS]) in the distribution of the trigeminal nerve, leptomeningeal angiomatosis and glaucoma [1]. The incidence of SWS is estimated to be 1 in 20,000 to 50,000 [2]. In 2013, Shirley and colleagues [3] found a GNAQ R183Q (c.548G>A) somatic mutation in both SWS and non-syndromic PWS patients. Since then, GNAQ mutation was confirmed in many SWS lesions. Recently, Bichsel et al. first discovered the GNAQ R183Q mutation in a case of SWS choroidal hemangioma and speculated that overabundant small vessels of choroid may contribute to increased intraocular pressure [4]. Choroidal vascular malformation is a frequent ocular manifestation in SWS patients. A study and literature review conducted in 1976 found that 20–70% of SWS patients suffered from choroidal hemangiomas [5]. Recent studies have shown thickening of the choroid in SWS patients by enhanced depth imaging optical coherence tomography (EDI-OCT) [6, 7]. However, due to the nature of the disease, the choroidal studies of SWS patients were all small case series. Moreover, a particular PWS distribution area, such as the ophthalmic branch of the trigeminal nerve, could be a predictor of SWS secondary glaucoma (SG) [8–12]. However, no studies have investigated the choroidal changes in non-glaucoma PWS (NGPWS) patients. Therefore, the aim of this study is to evaluate the choroidal changes in SG and NGPWS patients by EDI-OCT.

## Methods

### Subjects

This was a case control study that included consecutive PWS and SWS patients over 3 years old who were screened or treated at the Department of Ophthalmology, Shanghai Ninth People’s Hospital, from April 2017 to October 2017. A total of 79 participants were included in this study, including 52 minors under the age of 18.The study was approved by the institutional review board of the Ninth People’s Hospital Affiliated with the Shanghai Jiao Tong University School of Medicine. All patients were subjected to the following examinations: intraocular pressure (IOP), direct and indirect ophthalmoscope examinations, EDI spectral-domain OCT, fundus photography and type B ultrasound scans. The diagnosis of SWS-induced glaucoma was made by the same ophthalmologist (W. G.).

### Diagnosis of SWS

The main characteristics of SWS include ipsilateral leptomeningeal angiomatosis in the parietal-occipital lobe, unilateral facial PWS, and glaucoma. Notably, these signs are usually only partially manifested [13]. However, in this study, all the subjects suffered from facial PWS. All SWS patients had PWS and glaucoma.

Diagnosis of SWS-induced glaucoma: For patients with early onset glaucoma, the glaucoma diagnosis criteria were defined as follows (two or more required): ① the cup-to-disc ratio (C/D) of ipsilateral eyes with PWS was > 0.5 or obvious asymmetry was present in the C/D (0.2) for ipsilateral and fellow eyes; ② cornea diameter enlargement (diameter > 11 mm in newborns or > 12 mm within 12 months); ③ IOP > 21 mmHg or obvious asymmetry between the 2 eyes (> 6 mmHg). However, the IOP is among the least accurate and most variable of all the parameters measured when assessing a child for glaucoma.

For late onset glaucoma patients, in addition to IOP measurement and C/D observation, a visual field test and retinal nerve fiber layer assessment were also required for glaucoma diagnosis (if compliant), but corneal conditions were not considered diagnostic signs.

### EDI-OCT scan

All patients were imaged using a Heidelberg Spectralis OCT instrument (Heidelberg Engineering, Heidelberg, Germany) with enhanced depth imaging modality. Choroidal thickness was measured under the fovea. The measurement of choroidal thickness used a 1:1 μm model in the Heidelberg Spectralis OCT image analysis system. A vertical distance line was drawn from the retinal pigment epithelium to the choroidal-scleral interface under the fovea.

**Fig. 1:**
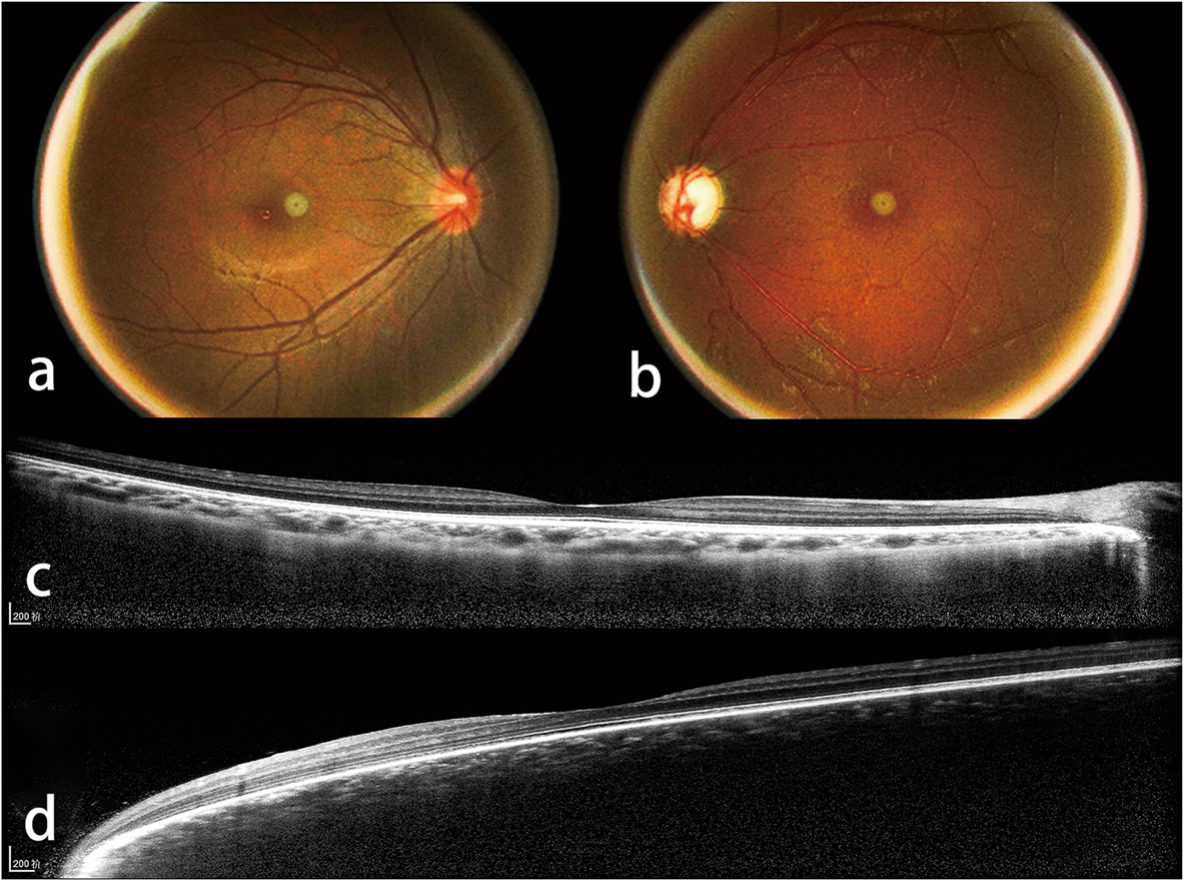
Color fundus photograph (**a**, **b**) and enhanced depth imaging optical coherence tomography (EDI-OCT) (**c**, **d**) of a Sturge-Weber syndrome secondary glaucoma patient shows the choroidal hemangioma criteria of the study. The color fundus photograph (**b**) shows a “ketchup” change in the left eye. EDI-OCT (**d**) of the left eye shows an elevation of the retina/choroid complex with a low-to-medium reflective signal from the lesion

Definition of choroidal hemangioma (both criteria had to be met): EDI-OCT showed elevation of the retina/choroid complex with a low-to-medium reflective signal from the lesion [14] and indirect ophthalmoscope and color fundus photography showed an orange elevated tumor [15] or a difference in color of the fundus between fellow eyes [13] (Fig. 1).

### Statistical analysis

All statistical analyses were performed with SPSS (Version 16, IBM Corporation, Armonk, NY, USA) and GraphPad Prism 8.0 (San Diego, CA). Data are summarized using the means and standard deviations or medians and interquartile ranges (IQR). Categorical data were analyzed with the Pearson χ2 test. Values were compared in the PWS and SWS groups using an independent T test. A *p* value less than 0.05 was considered statistically significant.

## Results

### Demographic data

Overall, 79 PWS patients were included in this study. Eyes ipsilateral to PWS were divided into NGPWS group (46) and SG group (35) according to the existence of ipsilateral glaucoma. Among these patients 2 exhibited bilateral PWS and unilateral glaucoma. Thus, the two eyes of each patient were divided into NGPWS and SG group, respectively. The mean age of NGPWS and SG were 16.52 ± 13.63 and 13.94 ± 8.27 years, respectively (*p* > 0.05). Among these SG patients, 3 had bilateral SWS-induced glaucoma, 32 had unilateral glaucoma. The IOP and C/D in ipsilateral eyes of SG patients with facial PWS were 24.58 ± 8.56 mmHg and 0.73 (0.64, 0.81), respectively, which significantly differed from those in fellow eyes (*p* < 0.0001). No significant differences were found between the ipsilateral and fellow eyes of NGPWS patients (*p* > 0.05) (Table 1).

**Table 1:** Baseline Demographic and Clinical Data

	NGPWS (case no. = 46)	SG (case no. = 35)
Sex		
Female	21	16
Male	25	19^b^
Age (yrs.), mean ± SD	16.52 ± 13.63	13.94 ± 8.27^c^
Location of the facial PWS		
Unilateral PWS	44	32^b^
Bilateral PWS	2	3
Glaucomatous eyes	0	38
IOP (mmHg), mean ± SD		
Ipsilateral eyes^a^	15.58 ± 3.57^c^	24.58 ± 8.56^d^
Contralateral eyes^a^	14.45 ± 3.43	15.13 ± 3.79
C/D, median (IQR)		
Ipsilateral eyes	0.30 (0.30, 0.40)^e^	0.73 (0.64, 0.81)^f^
Contralateral eyes	0.30 (0.30, 0.36)	0.30 (0.30, 0.40)
Simple excessive thickening of choroid eyes		
Ipsilateral eyes	2	5
Contralateral eyes	0	0
Choroidal hemangioma eyes	0	21^g^
Choroidal thickness (μm), mean ± SD		
Ipsilateral eyes	358.10 ± 117.40^h^ (45 eyes)	511.40 ± 242.10^h, i^(15 eyes)
Contralateral eyes	288.20 ± 79.04 (41 eyes)	283.90 ± 92.27^c^ (29 eyes)

*IOP* Intraocular pressure, *C/D* Cup-to-disc ratio, *IQR* Interquartile range, shown as (25, 75%)

^a.^ Ipsilateral eyes were the eyes in same side of the facial hemangiectasis while the contralateral eyes were in non-PWS side

^b.^ *p*>0.05, χ^2^ test

^c.^ *p*>0.05, unpaired *t* test

^d.^ *p*<0.0001, unpaired *t* test

^e.^ *p*>0.05, Mann-Whitney test

f. *p*<0.0001, Mann-Whitney test

g. *p*<0.0001, Fisher’s Exact Test

h. *p*<0.05, compared with the contralateral eyes using unpaired *t* test

i. *p*<0.05, compared with the ipsilateral eyes of NGPWS patients using unpaired *t* test

**Fig. 2:**
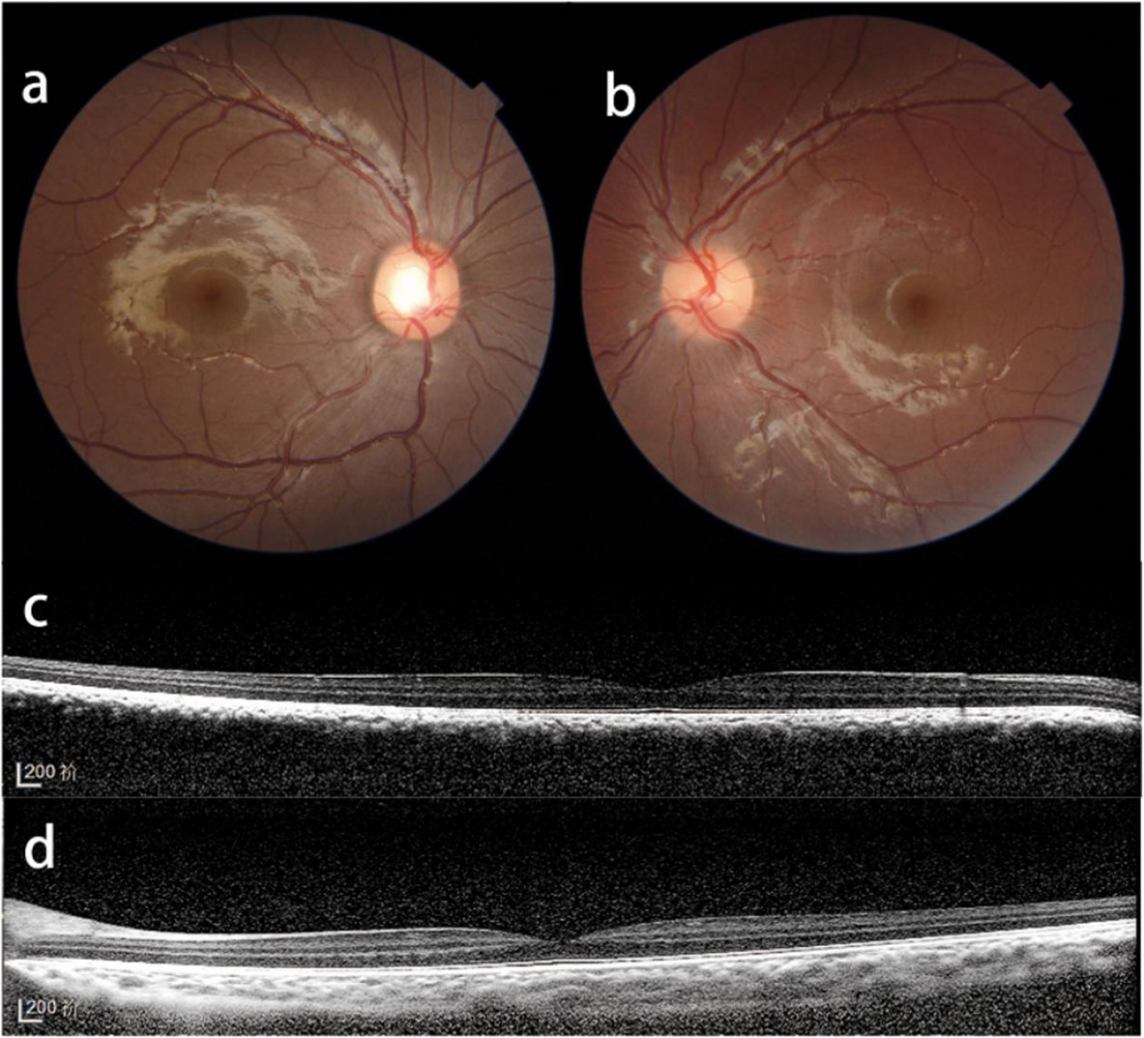
Color fundus photograph (**a**, **b**) and enhanced depth imaging optical coherence tomography (EDI-OCT) (**c**, **d**) of a Sturge-Weber syndrome secondary glaucoma patient shows excessive thickening of the choroid without choroidal hemangiomas. The color fundus photograph (**a**) shows no color changes compared to the fellow eye. EDI-OCT (**d**) shows no elevation of the retina/choroid complex but with a low-to-medium reflective signal from the lesion

EDI-OCT view of the choroid in NGPWS and SG patients: Among the patients, 21 eyes were diagnosed with choroidal hemangioma, which was only present in the SG eyes (55%). Additionally, 2 and 5 ipsilateral eyes of NGPWS and SG patients, respectively, exhibited a vague choroidal-scleral junction due to excessive thickness of the choroid. However, in these eyes, no retina/choroid complex elevation could be observed by EDI-OCT, while no orange elevated tumor in the posterior pole of the fundus could be found by indirect ophthalmoscope and fundus photography (Fig. 2). B-scan ultrasound showed diffuse choroid thickening. Thus, these 7 eyes were not diagnosed with choroidal hemangioma according to the criteria. Overall, the choroidal-scleral interface could not be examined in 28 eyes by EDI-OCT because 21 eyes had choroidal hemangiomas and 7 eyes had excessive thickening of the choroid without choroidal hemangiomas. The choroidal thickness could be measured by EDI-OCT in the remaining eyes. The choroidal thicknesses of the ipsilateral and fellow eyes of NGPWS group were 358.10 ± 117.40 μm (45 eyes) and 288.20 ± 79.04 μm (41 eyes), respectively (*p* < 0.05). The choroidal thicknesses of the ipsilateral and fellow eyes of SG group were 511.40 ± 242.10 μm (15 eyes) and 283.90 ± 92.27 μm (29 eyes), respectively (*p* < 0.05). Significant differences were observed between the ipsilateral eyes of SG and NGPWS patients (*p* < 0.05) (Table 1).

### Choroid and glaucoma in SWS patients

**Fig. 3:**
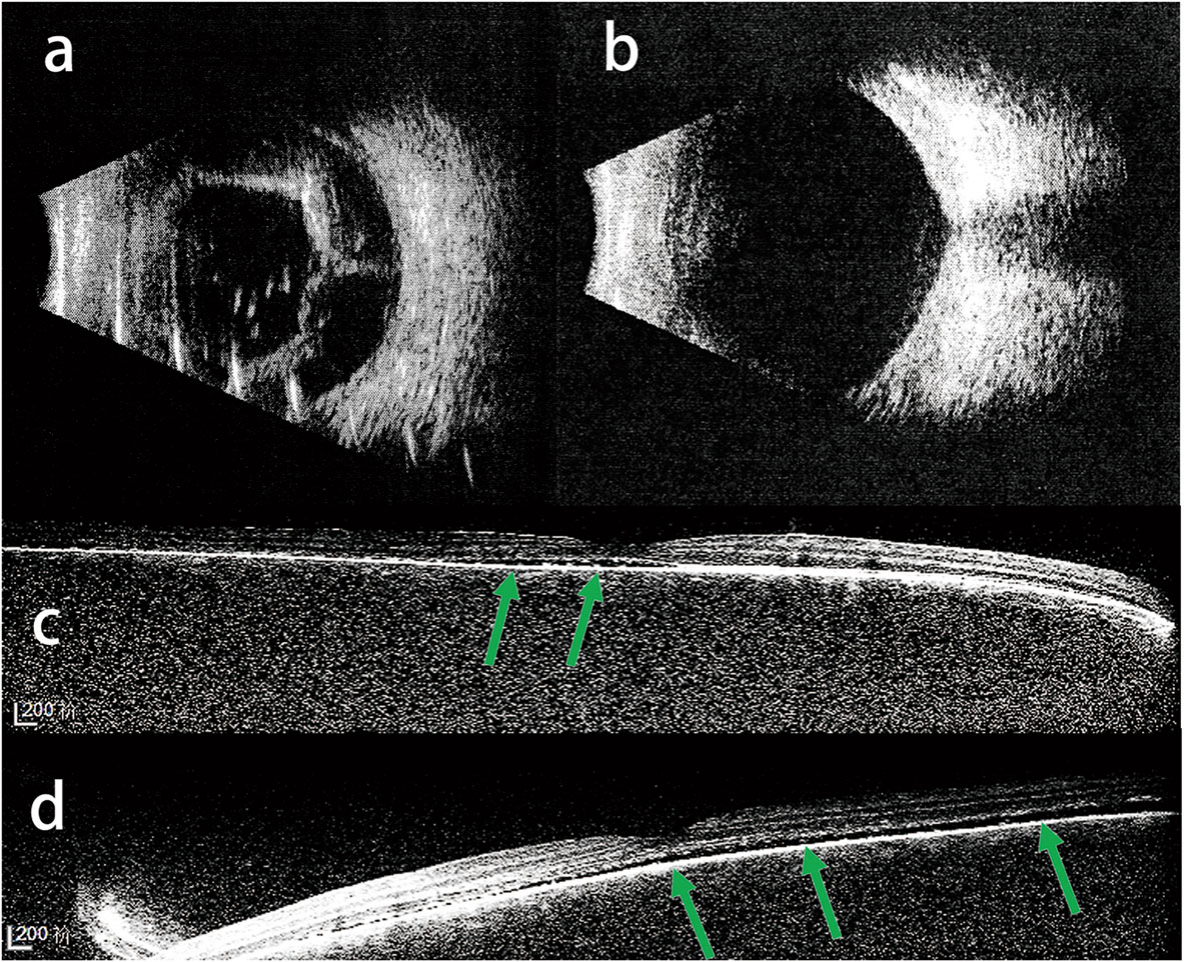
Type B ultrasound (**a**) shows retinal and choroidal detachment 1 week after Ex-PRESS implantation in an 8-year-old Sturge-Weber syndrome (SWS) secondary glaucoma patient. B-scan ultrasound (**b**) shows retinal and choroid recovery after 3 months of topical glucocorticoid and atropine administration. Enhanced depth imaging optical coherence tomography (EDI-OCT) (**c**, **d**) shows two eyes with mild retinal detachment (green arrow) in a 3-year-old bilateral SWS-induced glaucoma patient 1 week after trabeculotomy. The EDI-OCT scan quality is low due to the patient’s young age

A total of 38 eyes were diagnosed with SG, of which 21 also had choroid hemangiomas. Among the SG patients, 19 eyes underwent anti-glaucoma surgeries. Thirteen eyes underwent filtering surgeries including trabeculectomy (1 eye), Ex-PRESS glaucoma filtration device (Alcon Laboratories, Fort Worth, TX, USA) implantation (11 eyes) and valve implantation (1 eye). Six eyes underwent trabeculotomy. Among them, 6 eyes (46%) with choroidal hemangioma had posterior segment ocular complications, 4 of which underwent Ex-PRESS (P-50) implantation and also had retinal and choroidal detachment; the other 2 eyes underwent trabeculotomy and exhibited mild retinal detachment (Fig. 3). None of the eyes without choroidal hemangioma exhibited posterior segment ocular complications. Existence of choroidal hemangioma was a risk factor for post-operative posterior segment ocular complications (odds ratio = 2.000, 95% confidence interval, 1.136–3.522, *p* = 0.044) (Table 2).

**Table 2:** Relationship between Choroidal Hemangioma to Post-operational Posterior Segment Complications in SWS Induced Glaucoma Eyes

	Choroidal Hemangioma Eyes	Non-choroidal Hemangioma Eyes
Count of Eyes	21	17
Underwent anti-glaucoma surgery (eyes)	13	7
Post-operational posterior segment complications (eyes)^a^		
Choroidal and Retinal Detachment	4	0
Retinal Detachment	2	0

^a.^ Existence of choroidal hemangioma is a risk factor of post-operational posterior segment complications (Odd Ratio = 2.000, 95% confidence interval, 1.136–3.522, *p* = 0.044)

## Discussion

SWS is a neurocutaneous disorder characterized by a PWS that affects the skin in the distribution of the ophthalmic branch of the trigeminal nerve [3]. The choroid is one of the most important vascular alteration sites associated with SWS [13]. Previous studies have shown increased choroidal thickness in SWS patients. However, the incidence of choroidal hemangiomas was not clear due to the limited number of subjects [6, 7]. Although a study and literature review conducted in 1976 found that 20–70% of SWS patients suffered from choroidal hemangiomas [5], the findings are limited by the case numbers and the efficiency of the examination because the incidence range exhibited large variation. Thus, choroidal changes in SWS patients should be re-evaluated. Clinically, ophthalmoscopy shows a bright red or red–orange appearance of the fundus related to the increase in well-formed choroidal vessels, while hemangiomas appear as diffuse or localized areas with a dark red color and a “tomato ketchup” appearance [13]. In the current study, we defined the choroid not only by the “ketchup” color of the fundus [7], but also by an elevation of the retina/choroid complex with a low-to-medium reflective signal from the lesion on EDI-OCT. Thus, this could show direct visual evidence of choroid hemangiomas in SWS patients. However, EDI-OCT was not suitable for choroidal tumors that were > 1.0 mm in height and/or > 9.0 mm in diameter because the borders fell beyond the boundaries of detection [14]. In this study, in 21 eyes with choroidal hemangiomas and 7 eyes with a choroidal thickness > 1.0 mm of the outer border of the choroid could not be defined by EDI-OCT.

Because abnormal blood vessels in SWS and PWS patients are usually localized to a single region on one side of the body, a somatic mutation has been proposed to explain the etiology of SWS [16]. In 2013, Shirley and colleagues [3] found a GNAQ somatic mutation that affected 88 and 92% of tissue of SWS and PWS patients, respectively. In the current study, we found that both SG and NGPWS patients had an increased choroidal thickness. Nevertheless, the thickness of the choroid in SG patients was significantly greater than that in NGPWS patients, while a higher proportion of SG patients exhibited a vague choroidal-scleral junction due to excessive choroidal thickness. Choroidal hemangiomas were only found in SG eyes (55%). The results of our study might indicate that the GNAQ somatic mutation contributes to the choroid vessel dilation responsible for the thickening of the choroid. However, what induces the varying degrees of choroidal thickness in SG and NGPWS, whether choroidal thickness play a role in the development of glaucoma or is just a secondary response, as well as the possible reason for onset of choroidal hemangioma in SWS patients remain unclear.

The etiology of SWS-induced glaucoma has been attributed to elevated episcleral venous pressures in juvenile- or adult-onset glaucoma [17], whereas patients with early-onset glaucoma have anterior chamber angle anomalies [18, 19]. Thus, glaucoma can be difficult to control, even with combinations of various IOP-lowering medications. When medical management is unsuccessful, anti-glaucomatous surgical approaches have attempted to lower the IOP [20]. However, the surgical management of SWS-induced glaucoma is challenging for ophthalmologists, not only because of the low surgical success rate but also due to severe post-operative complications, such as choroidal effusion, choroidal detachment and exudative retinal detachment [21–24]. Post-operative complications of the posterior segment mostly occurred after filtering surgeries, possibly due to relieving the eye pressure too quickly [20]. In this study, 19 eyes underwent anti-glaucoma surgery. Among them, 13 underwent filtering surgery, including trabeculectomy (1 eye), Ex-PRESS implantation (11 eyes) and valve implantation (1 eye). Six eyes exhibited posterior segment ocular complications, including 4 with retinal and choroidal detachment and 2 with mild retinal detachment. All of these complications occurred in eyes with choroidal hemangioma. Most notably, 2 eyes (1 patient) were found to have mild retinal detachment by EDI-OCT after trabeculotomy. As a non-filtering surgery mostly applied to infant patients, trabeculotomy is believed to be safe and effective for children. Ikeda and colleagues [25] found that 3.4% of eyes exhibited retinal detachment during developmental glaucoma after trabeculotomy due to an enlarged globe. However, previous studies did not find posterior segment complications in SWS patients [26, 27]. In our study, the axial lengths in the two eyes were 24.2 mm and 23.1 mm. The globe was enlarged for the 3-year-old child underwent trabeculotomy in the present study [28]. However, it was worth noting that this patient had two eyes with choroidal hemangiomas. According to the pattern of the retinal detachment and the lack of a retinal hole, we were inclined to believe that the posterior segment complication was due to exudative retinal detachment. A previous study also showed exudative retinal detachment after a strabismus surgery in an SWS patient [29]. This indicates that only a moderate change of IOP or manipulation of the eyeball could disturb choroidal hemangiomas and increase the effusion. It is worth noting that post-operative choroidal detachment and exudative retinal detachment in SWS-induced glaucoma patients were typically self-limiting, with all 6 eyes recovering within 3 months of topical glucocorticoid and atropine administration.

In addition, our previous work demonstrated that different distribution patterns of episcleral hemangioma lead to different trabeculotomy prognosis in young SG patients [30]. Compared to episcleral vascular malformations, choroidal hemangioma may not be a pathogenic factor in SG. But SG patients did have a higher proportion of choroidal hemangioma and excessively thickened choroid, which may indicate the distinct choroidal alteration in NGPWS eyes and SG eyes. Further study on the pathological alterations of the choroid in SWS patients and clarification of the relationship between choroidal hemangioma and episcleral hemangioma distributions patterns is warranted.

The present study has some limitations. In some patients with PWS magnetic resonance imaging of the central nervous system could not be performed due to age of patients, economic factors, and psychosocial issues. Thus, only 4 patients were confirmed as epileptic seizures. PWS patients that only have moderate leptomeningeal angiomatosis but no sign of epileptic seizure may exist in our study.

## Conclusions

NGPWS and SG patients had a greater choroidal thickness in the ipsilateral eye than in the fellow eye. The trend was even more pronounced in SG patients. All choroidal hemangiomas were found in the ipsilateral eyes of SG patients with facial PWS. In addition, choroidal hemangioma was a risk factor for post-operative complications of the posterior segment in SWS patients, even if the anti-glaucoma surgery was a trabeculotomy. Thus, fundus observation for choroid alterations in SWS patients using EDI-OCT was useful for evaluating the risk of the anti-glaucoma surgery.

## Data Availability

Most of data generated or analyzed during this study are included in this published article and in its supplementary information files. The remaining datasets are available from the corresponding author upon reasonable request.

## Abbreviations

C/D: Cup-to-disc ratio
IOP: Intraocular pressure
NGPWS: Non-glaucoma port-wine stain
PWS: Port-wine stain
SWS: Sturge-Weber syndrome
SG: Sturge-Weber syndrome secondary glaucoma
